# Effect of temperature on capping efficiency of zeolite and activated carbon under fabric mats for interrupting nutrient release from sediments

**DOI:** 10.1038/s41598-019-52393-1

**Published:** 2019-10-31

**Authors:** Seung-Hee Hong, Jae-In Lee, Chang-Gu Lee, Seong-Jik Park

**Affiliations:** 1 Department of Bioresources and Rural System Engineering, Hankyong National University, Anseong, South Korea; 2 Department of Environmental and Safety Engineering, Ajou University, Suwon, South Korea

**Keywords:** Environmental sciences, Limnology

## Abstract

We investigated the influence of temperature on the capping efficiency to interrupt the release of nutrients from lake sediments. A 3-cm layer of Zeolite (ZL) or activated carbon (AC) was placed on the contaminated sediments, and nonwoven fabric mats (NWFM) were placed on top of these capping materials. Laboratory incubation experiments were performed under three different temperatures, namely 4, 15, and 30 °C. Under the uncapped condition at 30 °C, dissolved oxygen (DO) was depleted after 30 days, while at 4 °C and 15 °C, DO was present until the end of this experiment. DO concentration in overlying water was more dependent on the temperature than capping condition. ZL/NWFM effectively blocked the release of N from the sediments, and the capping efficiencies of ZL/NWFM for NH_4_-N at 4, 15, and 30 °C were 98%, 96%, and 94%, respectively. For the interruption of P release, both ZL/NWFM and AC/NWFM were not effective at 4 and 15 °C. At 30 °C, however, AC/NWFM was effective, and its capping efficiencies at 30 °C for PO_4_-P and T-P were 74.0% and 79.9%, respectively. In summary, nutrient release from sediments was accelerated at higher temperatures, and the effect of capping was significant at high temperature.

## Introduction

Along with monitored natural recovery and dredging, *in-situ* capping is the most widely used method for the remediation of contaminated sediments. Placing a cap layer on the contaminated sediments minimizes resuspension and transport of sediment particles, stabilizes sediments, and reduces the diffusion of dissolved contaminants into the overlying waters^1^. Approximately 50-cm thickness of sand or gravel was placed on the top of contaminated sediment as a cap layer, in order to isolate it physically from the overlying water^2^. A demonstration test (100 m × 100 m) for capping was conducted by placing a layer of clean coarse sand on the sediments in Hamilton Harbor, Lake Ontario, Canada during the summer of 1995^3^. To improve the effectiveness of capping for specific contaminants, Jacobs and Förstner^4^ suggested capping contaminated sediments with an active barrier system, in which geochemical materials that can bind contaminants via adsorption or precipitation processes are used. Murphy *et al*.^5^ evaluated the effectiveness of applying a thin layer (1.25 cm) of an active sorbent like organic-rich soil, coke, or activated carbon. Thin layer capping has attracted the interest of many researchers in recent years.

Thin layer capping has recently been the focus of intensive studies for the remediation of sediments contaminated with nutrients, especially NH_4_^+^ and P. Various kinds of capping materials have been extensively investigated. However, the influence of environmental conditions on the capping efficiency has received relatively little attention. Lanthanum modified bentonite clay and Bauxsol were assessed as capping agents for reducing the release of P from lake sediments during anoxic/oxic cycles^6^. Thermally-treated calcium-rich attapulgite was evaluated as a P-inactivation agent for lake eutrophication control under different pH conditions and in the presence of co-existing ions^7^. Zou *et al*.^8^ used synthesized ferrihydrite as a capping layer for sequestrating P in wetland sediment by alternating the oxic and anoxic conditions using laboratory reactors. Xiong *et al*.^9^ investigated the influence of the Na^+^/Ca^2+^ concentration and redox potential of the overlying water on the release of NH_4_^+^, PO_4_^3−^, and heavy metals from sediments capped with natural zeolite. Most of these studies performed incubation experiments by monitoring the contaminant concentration in the overlying water or sediments at room temperature, or other constant temperature^10–12^. To the best of our knowledge, the effect of temperature on the efficiency of capping materials for the interruption of release of contaminants from sediments has never been addressed.

In temperate and subpolar regions, the four seasons are distinct and span a wide range of temperatures. In Korea, the average temperature in August is ~23–26 °C, while that of January is ~−6–3 °C. The annual average temperature is ~10–15 °C. A wide range of temperatures throughout the year can influence the temperature of the sediment in lakes, particularly in the case of shallow lakes. Along with the dissolved oxygen in the water, temperature has been considered as the most important factor resulting in microbiological processes in sediments, leading to nutrient release^13^. The increase in temperature enhances the activity of bacteria, benthic algae, and phytoplankton^14^. The enhancement of microbial activity in sediments by the increase in temperature can lead to a change in environmental conditions and the release of contaminants from sediments into the overlying water. P release was reported to be dependent on temperature and clearly associated with the microbial activity in sediments^15,16^.

In the present study, we selected zeolite and activated carbon as materials for interrupting the release of nutrients from contaminated sediments. Zeolite has proven to be effective in interrupting the release of cationic contaminants such as NH_4_-N and heavy metals^12^. However, zeolite has some limitations, including its inefficiency in sequestrating anionic phosphate and chromate^12,17^. To fix P in sediments along with other cationic contaminants, zeolite modified with metals such as Al, Fe, La, and Zr has been proposed by other studies^17–19^. Along with zeolite, active carbon has been widely used as capping material, especially for sequestrating organic contaminants. Activated carbon has many advantages as capping material, such as huge specific surface area for binding contaminants, less public concern for its application, and proof-of-concept of its use in many studies^20,21^. In our previous study^20^, the use of non-woven fabric mats with activated carbon prevented the loss of activated carbon by flotation and external forces, which proved to be more effective in sequestrating N and P than the use of activated carbon alone.

The aim of this study is to investigate the influence of temperature on the release of contaminants from sediments and the efficiency of zeolite (ZL) and activated carbon (AC) under non-woven fabric mats (NWFM) in terms of interrupting this release. The capping efficiency was evaluated by performing column incubation experiments for 60 days at three different temperatures, i.e., 4, 15, and 30 °C, where these temperatures were manipulated to mimic seasonal variations. Environmental parameters including dissolved oxygen (DO), pH, the oxidation reduction potential (ORP), and electric conductivity (EC), as well as the concentration of nutrients including chemical oxygen demand (COD), NH_4_-N, NO_3_-N, total N (T-N), PO_4_-P, and total P (T-P) were monitored in the overlying water. The flux of N and P and the capping efficiency were calculated, and the effects of temperature and capping materials on the flux of nutrients were statistically analyzed.

## Results and Discussion

### Contaminant release from sediment at different temperatures

The physicochemical characteristics of water and sediments sampled from the lake are shown in Table 1. T-N and T-P concentration in water were 1.88 mg/L and 0.18 mg/L, respectively, corresponding to Level 6 (worst water quality level) according to the water quality standards for lakes established by the Korean Ministry of Environment. The T-N content in sediments was 1227.4 mg/kg, corresponding to moderate pollution (1000–2000 mg/kg) according to US EPA guidelines, even though this value is below the assigned sediment quality guidelines of the Korean Ministry of Environment. The T-P content in sediments was likewise below the Korean sediment quality threshold but exceeded high level concentrations (>650 mg/kg) according to US EPA guidelines. These results indicate that the water and sediments in the lake investigated in this study are highly eutrophic. The COD concentration in the water corresponded to Level 6 according to the Korean water quality standards, and the COD content in sediments was within the moderate pollution range (40,000–80,000 mg/kg), according to US EPA sediment criteria. These high concentrations of C, N, and P are due to pollution from a high density of pig farms in the areas near the lake and surface runoff from inclined upland farms.

**Table 1:** Properties of water and sediment sampled from Mansu Lake in January 2018.

Water		Sediment	
Property	value	Property	Value
pH	8.06	pH	7.48
EC (µS/cm)	294.50	EC (µS/cm)	530.0
DO (mg/L)	8.33	Organic matter (%)	10.7
SS (mg/L)	605	Water contents (%)	70.3
ORP (mV)	−53.3		
T-N (mg/L)	1.88	T-N (mg/kg)	1235.0
NH_4_-N (mg/L)	0.23	NH_3_-N (mg/kg)	26.3
NO_3_-N (mg/L)	0.28	NO_3_-N(mg/kg)	2.5
T-P (mg/L)	0.18	T-P (mg/kg)	1507.9
PO_4_-P (mg/L)	0.08	P_2_0_5_ (mg/kg)	250.0
COD (mg/L)	26.6	COD (mg/kg)	59010.0

### Effect of temperature and capping on water environment

**Figure 1:**
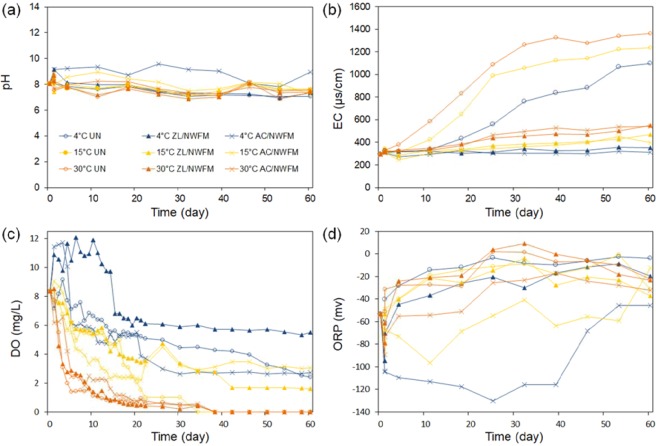
Changes in water environmental conditions during 60 days of laboratory incubations. (**a**) pH, (**b**) electric conductivity (μS/cm), (**c**) dissolved oxygen (mg/L), (**d**) oxidation reduction potential (mV). These were obtained by the analysis of overlying water.

Figure 1 illustrates variations in pH, EC, DO, and ORP in the water overlying uncapped and capped sediments during 60 days experiments. The pH of the overlying water with AC/NWFM capping at 4 °C and 15 °C was remarkably different from the pH values obtained under other experimental conditions, which ranged from 7 to 8. The increase in pH with the AC/NWFM capping can be explained primarily by the formation of alkaline ash during the manufacturing process. Inorganic materials such as Ca, Na, and K in the raw AC material are altered into Ca(OH)_2_, NaOH, and KOH, respectively, by reacting with O_2_ and H_2_O during the activation process^20^. Higher temperatures enhance the elution of such alkaline elements of AC into the overlying water, which results in the increase of pH with temperature for AC/NWFM capping. The EC in the overlying water under all experimental conditions increased with time along the experimental run. The EC under the uncapped condition was higher than that in capping conditions. Moreover, the EC at 30 °C was higher than that of the lower temperatures. These results indicate that a higher amount of soluble ions were released from the sediments under uncapped conditions and at higher temperatures during the experiment.

In contrast to EC, the concentration of DO in the overlying water decreased over time under all experimental conditions. At 30 °C, DO concentration dropped sharply within 7 days and continued its decreasing trend thereafter, to be exhausted after 40 days. The decrease of DO concentration over time was also observed at 4 °C and 15 °C, but their decreases were smaller than that of 30 °C. The average and standard deviation of DO (mg/L) in the overlying water are given in decreasing order as follows: 4 °C-ZL/NWFM (8.12 ± 2.43) > 4 °C-UN (5.66 ± 1.67) > 4 °C-AC/NWFM (5.30 ± 2.65) > 15 °C-ZL/NWFM (4.49 ± 2.00) > 15 °C-AC/NWFM (3.97 ± 2.00) > 15 °C-UN (3.74 ± 2.68) > 30 °C-AC/NWFM (1.90 ± 2.13) > 30 °C-ZL/NWFM (1.54 ± 2.11) ≈ 30 °C-UN (1.40 ± 2.01). The DO concentration in overlying water was more dependent on the temperature than capping condition. DO concentrations in the case of ZL/NWFM capping at same temperature were higher than those in uncapped and AC/NWFM capping conditions, indicating that ZL/NWFM capping delayed the consumption of DO in the overlying water. This result can be explained by that organic matter and NH_4_^+^, which consume DO in overlying water through nitrification, was effectively captured by ZL^22^. The ORP (Eh) decreased during the early stage of the experiment, and thereupon continuously increased to ~−4.2 mV or ~−45.9 mV, depending on the capping condition. This result is consistent with the results in other studies^20,23,24^, which reported that *Eh* decreases for the first few days after soil is submerged, then turns to an increasing trend. The ORP under AC/NWFM capping condition was much lower than that of other experimental conditions, indicating that stronger reduction conditions were formed under AC/NWFM capping condition. This could be due to the presence of organic functional groups on the surface of AC/NWFM, including phenolic, carboxyl, and quinone, which act as reducing agents^25^.

### Effect of temperature and capping on the release of nitrogen and phosphorus

**Figure 2:**
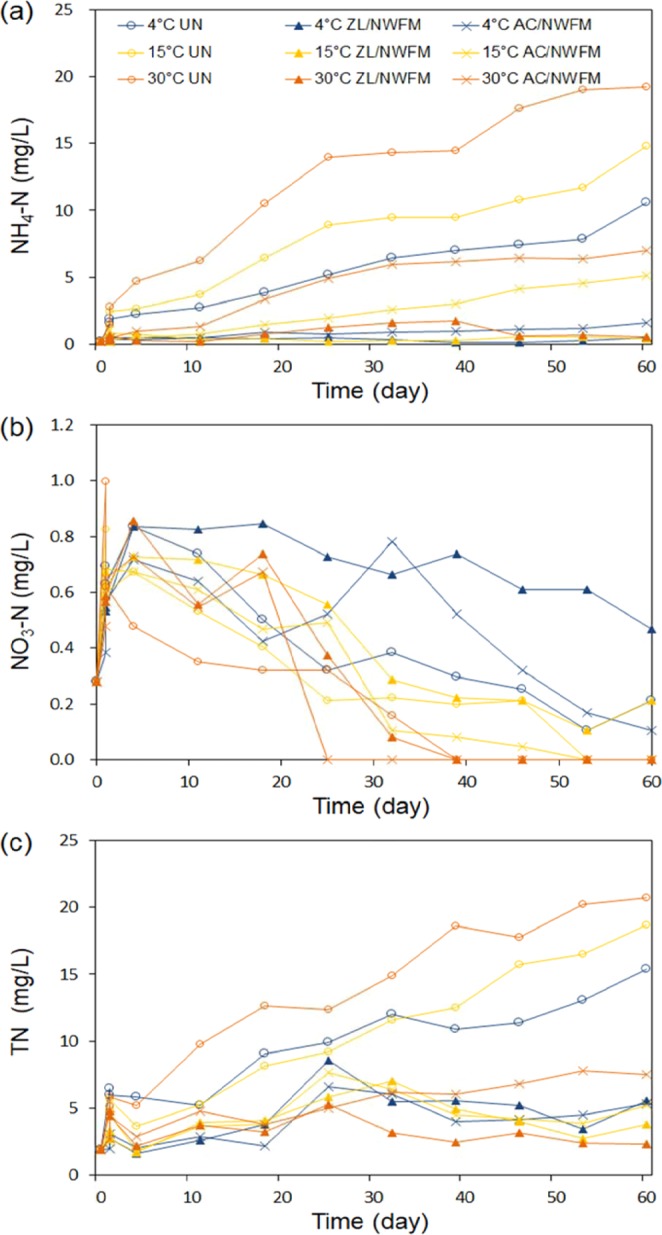
Changes in nitrogen concentration at different temperatures and capping conditions during 60 days of laboratory incubations. (**a**) NH_4_-N (mg/L), (**b**) NO_3_-N (mg/L), (**c**) T-N (mg/L).

The dependence of various N, i.e., NH_4_-N, NO_3_-N, and T-N concentrations in the water overlying the sediments on temperature and capping materials with respect to time is shown in Fig. 2. Under the uncapped condition, the NH_4_-N concentrations at 4, 15, and 30 °C increased continuously with time to reach 10.6 mg/L, 14.8 mg/L, and 19.2 mg/L, respectively (Fig. 2a). The NH_4_-N concentration of overlying water in AC/NWFM capping condition was also highly dependent on temperature, and likewise increased with time. ZL/NWFM capping demonstrated a lower NH_4_-N concentration than other capping conditions. By According to Stokes-Einstein equation^26^, the diffusion coefficient at 30 °C is about 10% larger than the value at 4 °C, indicating that the contribution of diffusion increases with temperature elevation. However, it is not likely that this small increase in diffusion has affected the concentration of soluble ions in overlying water significantly. Larger amount of ammonium released at higher temperature was mainly caused by the enhanced decomposition rate of organic nitrogen to ammonia via biological process^27^. Within the optimal temperature range, the biological processes increase two-fold for each 10 °C rise in temperature^28^. The optimum temperature for organic nitrogen mineralization is higher than 35 °C, indicating that the rate of organic nitrogen mineralization can increase up to this temperature^29^. Biological activity also influences the sediment oxygen demand, and high temperature also accelerates the depletion of oxygen in overlying water, which leads to anaerobic condition^27^.

**Figure 3:**
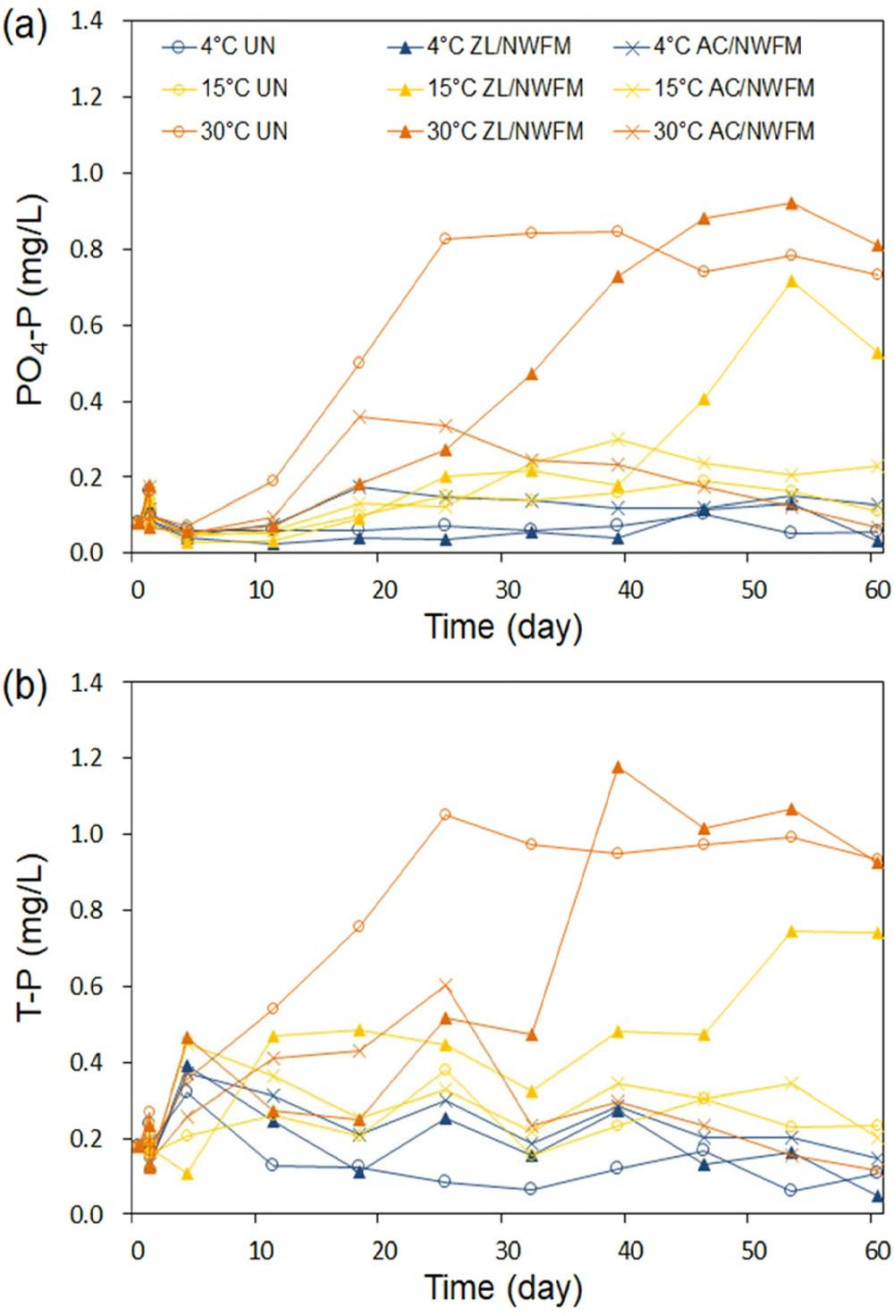
Changes in (**a**) PO_4_-P and (**b**) T-P concentration at different temperatures and capping conditions during 60 days of laboratory incubations.

In contrast to NH_4_-N, the NO_3_-N concentration in all experimental conditions deceased with time, finally disappearing at 30 °C within 40 days, under both uncapped and capped conditions. The inverse correlation between the release of NH_4_-N and NO_3_-N was also observed in the study of Liikanen *et al*.^13^. The disappearing NO_3_-N concentration in this study can be explained by the depletion of DO in water at 30 °C on day 39 (Fig. 3a). The NO_3_^−^ reduction under anaerobic conditions occurs by the denitrification process^30^. The lower DO concentrations at 30 °C in comparison to those of other temperatures also contributed to not only the reduction of NO_3_^−^, but also to the increase in the NH_4_^+^ concentration of overlying water. In anaerobic bottom-water conditions, the biological oxidation of ammonium to nitrate, i.e., nitrification, is inhibited^12,31^. Therefore, the dissolved inorganic N released from the sediments appears as NH_4_^+^.

The change in T-N concentration with time exhibits similarities to the NH_4_-N concentration change, and their concentrations share a similar range. This result infers that T-N is mainly composed of NH_4_-N, and that NH_4_-N is the dominant form of N released from the sediments under low oxygen concentrations. This result is consistent with the one in our previous research^20^, where NH_4_-N occupied 90% of T-N in the water overlying the uncapped sediments. Beutel^31^ also reported that N was released from sediments to the overlying water mainly in the form of NH_4_^+^ after the decomposition of organic N in sediments.

The change in PO_4_-P and T-P concentrations with time at different temperatures and capping conditions is shown in Fig. 3. In uncapped sediments at 30 °C, PO_4_-P and T-P concentrations continuously increased, reaching 0.83 mg/L and 1.05 mg/L, respectively, within 25 days, and those concentrations were kept constant. The increase in PO_4_-P and T-P concentrations within 25 days is consistent with the sudden decrease of DO concentration at start of incubation. The PO_4_-P concentration in the uncapped condition significantly differed depending on the temperature, and such a discrepancy was more pronounced toward the end of the experiments (anoxic condition) in comparison to the beginning of experiments (oxic condition). This result was also observed by Liikanen *et al*.^13^, in whose study the temperature did not correlate with the P release under oxic conditions, but the rate of P release under anoxic conditions was more dependent on the organic matter mineralization regulated by temperature. Increasing the respiration rate of bacteria in the sediment at higher temperatures accelerates DO depletion, which facilitates the occurrence of a low redox potential. The low redox potential induces the reduction of Fe^3+^ to Fe^2+^ and finally results in Fe/Al-P release, with an increase of dissolved P in the overlying water^14^.

### Flux and capping efficiency under different temperatures and capping materials

**Figure 4:**
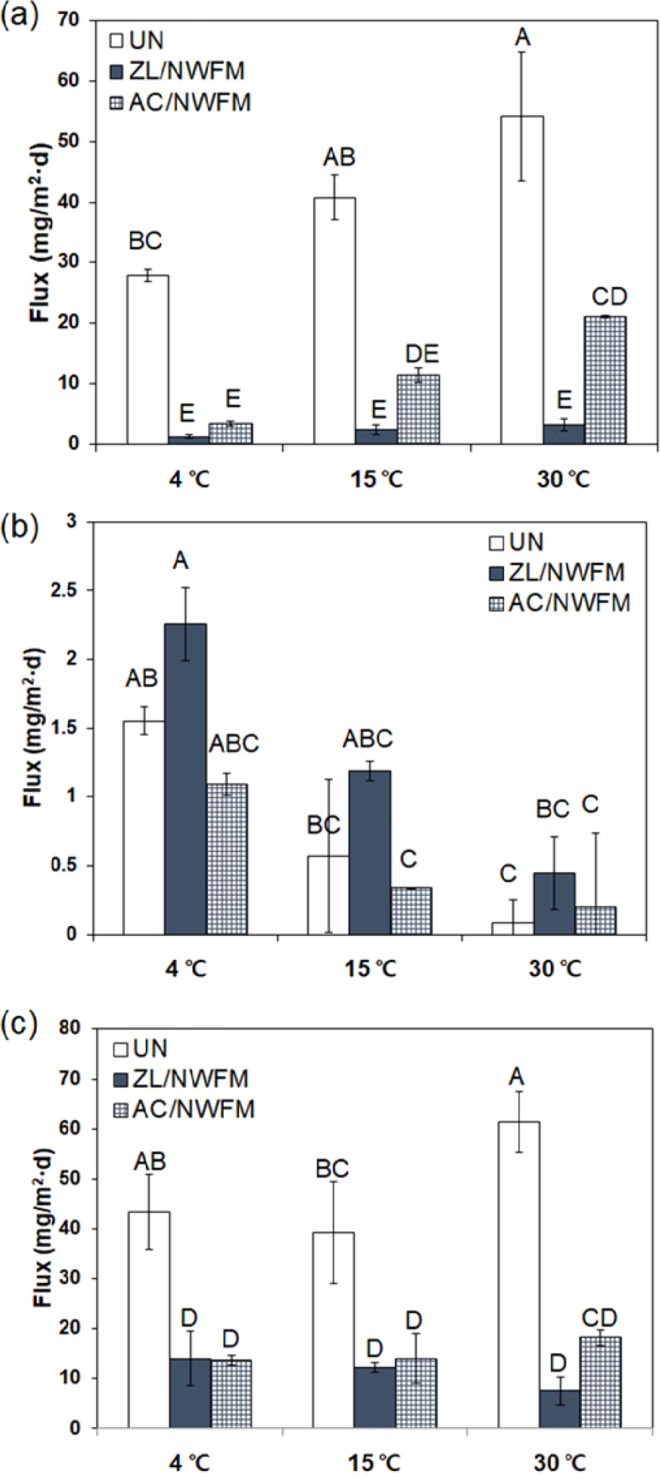
Comparison of fluxes of (**a**) NH_4_-N, (**b**) NO_3_-N, and (**c**) T-N in uncapped sediments and sediments capped with zeolite and nonwoven fabric mats, as well as activated carbon and nonwoven fabric mats, at different temperatures. The letters above the bars depict the significant differences according to Tukey’s multiple range test (*p* < 0.05). Capping efficiency was calculated using Eq. 1 for 60 days of incubation experiments. The error bar represents the standard deviation of two sets of experiments.

The fluxes of N including NH_4_-N, NO_3_-N, and T-N from the sediment into the overlying water at different temperatures and capping conditions were calculated, as shown in Fig. 4. The NH_4_-N flux depicted in Table 2 demonstrated significant differences depending on both capping materials (*p* < 0.001) and temperatures (*p* < 0.001). A higher NH_4_-N flux under the uncapped condition was observed with the increase of temperature. The NH_4_-N flux under AC/NWFM capping condition also increased from 3.4 mg/m^2^·d to 21.1 mg/m^2^·d as the temperature increased from 4 °C to 30 °C. However, the change in the NH_4_-N flux in the sediments capped with ZL/NWFM with increase in temperature was statistically insignificant. The capping efficiencies of ZL/NWFM for interrupting the release of NH_4_-N at 4, 15, and 30 °C were 95.4%, 94.1% and 94.1%, respectively. The amount of NH_4_^+^ released from sediments increased along with temperature; but it was effectively interrupted by ZL/NWFM capping. The adsorption of NH_4_^+^ onto ZL is an endothermic process, and the efficiency of ZL/NWFM capping can be enhanced by elevated temperature^32^. In contrast to ZL/NWFM capping, the capping efficiency of AC/NWFM was dependent on temperature, and it was decreased from 88.1% to 61.1% as the temperature increased from 4 °C to 30 °C. Due to its lower adsorption capacity for NH_4_^+^, AC could not effectively interrupt the increased release of NH_4_^+^ upon temperature elevation.

**Table 2:** Summarized results of two-way ANOVA (material and temperature) of nitrogen and P fluxes from sediments to overlying water.

Source	df	Flux (*p*)				
		NH_4_-N	NO_3_-N	T-N	PO_4_-P	T-P
Model	8	<0.001	0.001	<0.001	<0.001	<0.001
Material	2	<0.001	0.005	<0.001	0.042	0.002
Temperature	2	<0.001	<0.001	0.109	<0.001	<0.001
Material * Temperature	4	0.018	0.361	0.043	0.001	<0.001

In contrast to NH_4_-N, a higher NO_3_-N flux was observed under the ZL/NWFM capping condition than in the uncapped condition. NO_3_-N flux decreased with increasing temperature under all capping conditions. This result is consistent with the fact that DO concentration under the ZL/NWFM capping condition was higher than other capping conditions at the same temperature. Because of such higher DO concentration in overlying water, higher NO_3_-N but lower NH_4_-N fluxes were observed in ZL/NWFM capping condition than other capping conditions. The magnitude of the NO_3_-N flux was lower by one order than the NH_4_-N flux and the contribution of the NH_4_-N flux to T-N flux can be larger than that of the NO_3_-N flux.

The T-N flux in the uncapped condition at 30 °C was higher than that at other temperatures, and T-N fluxes under ZL/NWFM capping condition were independent of the temperature. In contrast to the NH_4_-N flux, the temperature did not significantly influence T-N fluxes (*p* = 0.109, shown in Table 2). Although the NH_4_-N flux under ZL/NWFM capping condition was significantly lower than under AC/NWFM capping condition, the difference between the T-N flux under ZL/NWFM capping condition and AC/NWFM capping condition was not significant at 4 °C and 15 °C. From this result, we can infer that the AC/NWFM capping is efficient with respect to interrupting the release of organic N from sediments, while ZL/NWFM capping is efficient in the same role for NH_4_^+^. ZL/NWFM can remove NH_4_^+^ through cation exchange between NH_4_^+^ in the overlying water and exchangeable cations adsorbed on the framework, formed from SiO_4_ and AlO_4_ tetrahedra of ZL^33^. Other studies observed that ZL represents the best barrier for the interruption of NH_4_^+^ from the sediments in both fresh water and seawater^9,12,34,35^. AC can effectively adsorb organic N and the N adsorbed on the particles because of its strong adsorption capacity for hydrophobic substances^36^.

**Figure 5:**
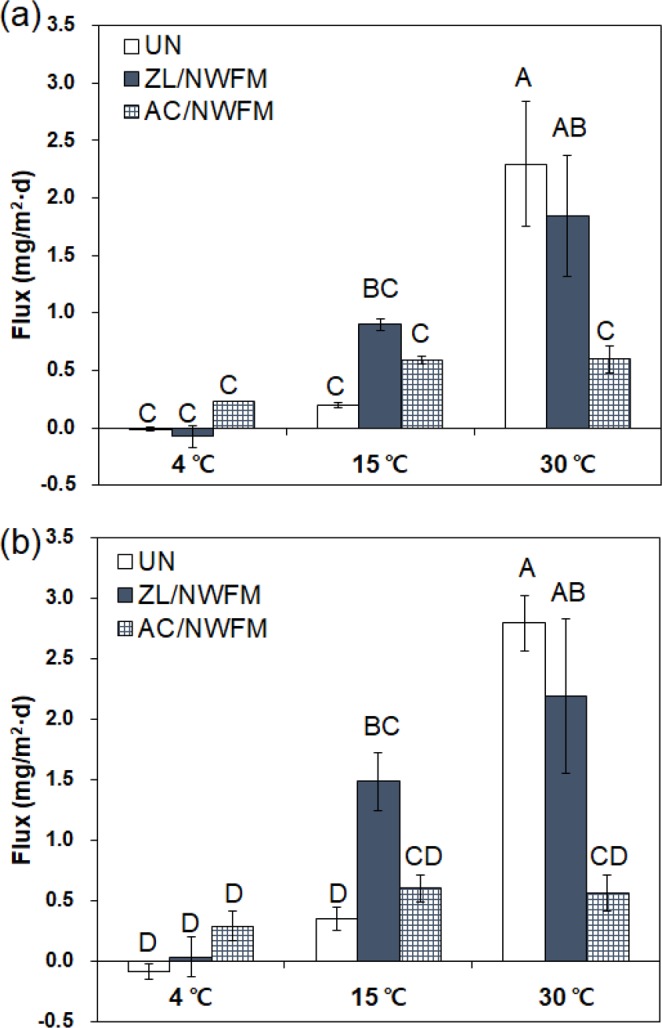
Comparison of fluxes (**a**) PO4-P and (**b**) T-P in uncapped sediments and sediments capped with ZL with NWFM, and AC with NWFM, at different temperatures. The letters above the bars depict significant differences according to Tukey’s multiple range test (p < 0.05). Capping efficiency was calculated using Eq. 1 for 60 days of incubation experiments. The error bar represents the standard deviation of two sets of experiments.

The fluxes of PO_4_-P and T-P at different temperatures and capping conditions were calculated and shown in Fig. 5. In the uncapped condition, the PO_4_-P and T-P fluxes at 4 °C were −0.008 and −0.088 mg/m^2^·d, respectively, and those at 30 °C were 2.296 and 2.800 mg/m^2^·d, respectively, indicating that the uncapped sediment acted a sink for P at 4 °C but a source at 30 °C. The higher amount of P release from sediments at higher temperatures is in agreement with a number of recent studies, which claim that sediment P release was observed in the (warmer) summer, while sediment P uptake was observed in the (cooler) winter months^37,38^. The magnitude of the P flux from sediments to the overlying water was less than that of N flux, which is consistent with the study of Wu *et al*.^39^, who reported that P exchange between the sediment and overlying water was less intensive than N. These results also indicate that N can act as an important contamination source to assess water quality.

In Table 2, PO_4_-P fluxes exhibited a slightly significant degree of variation with respect to the capping material, while they were significantly influenced by the temperature. At 4 °C, the fluxes of both PO_4_-P and T-P did not show significant differences with respect to the capping conditions. At 15 °C, the fluxes of PO_4_-P and T-P under ZL/NWFM capping and AC/NWFM capping conditions were not statistically lower than those under uncapped conditions, indicating that ZL/NWFM and AC/NWFM were not efficient for interrupting release of PO_4_-P and T-P at 15 °C. However, lower fluxes of PO_4_-P and T-P at 30 °C were observed under ZL/NWFM capping condition and AC/NWFM capping condition in comparison to uncapped conditions. Although the ZL/NWFM capping was more efficient for interrupting the release of NH_4_-N than AC/NWFM capping, lower fluxes of PO_4_-P and T-P were observed under AC/NWFM capping condition. The capping efficiencies of ZL/NWFM capping for PO_4_-P and T-P at 30 °C were 19.6% and 21.6%, respectively, and those of AC/NWFM capping were 74.0% and 79.9%, respectively. At 15 °C and 30 °C, the ratio of PO_4_-P flux to T-P flux in AC/NWFM capping was near 100%, much higher than that in uncapped sediments (~57.7%–82.1%) and in ZL/NWFM capping (~60.6%–84.2%), indicating that the release of P from the sediments capped with AC/NWFM mainly occurs in the form of dissolved inorganic P. Therefore, we speculate that AC/NWFM capping reduces the P concentration in overlying water by preventing the resuspension of particulates onto which P is adsorbed, due to the hydrophobic nature of AC. However, AC is less effective in interrupting the release of dissolved inorganic P.

The important observations in this study comprise the increase of the release of N and P from uncapped sediments at higher temperatures. The higher temperature provided favorable conditions for the consumption of DO in the overlying water. Distinction of DO in the overlying water leads to the release of NH_4_-N and P from sediments into the overlying water. High concentrations of NH_4_-N and P in the overlying water can trigger algal bloom. Algal bloom may lead to the reduction of DO, causing a vicious circle for deterioration of water quality and damaging the health of the ecosystem. Some of the results not investigated in this study, but reported in other literature, indicate that the oxygen deficiency in sediments and water contribute to the production of the greenhouse gas, CH_4_, which increases the global warming potential^13^. Consequential to the increase in nutrient release from the sediments and the associated increase in primary production and oxygen deficiency, the CH_4_ emissions would increase more than predicted by the temperature increase alone. Interrupting the release of N and P from sediments using capping technologies presents the way to break this vicious circle.

## Conclusions

In this study, we investigated the influence of temperature on the capping efficiency of ZL and AC capping with NWFM. Lower DO concentrations in the overlying water were observed at higher temperatures, and DO under all capping conditions was depleted at 30 °C. Higher amounts of NH_4_-N were released from the sediments with increasing temperature due to the depletion of DO in the overlying water. The tendency of NH_4_-N release was similar to the T-N release, indicating that N is released mainly in the form of NH_4_^+^. PO_4_-P release was dependent on the temperature under the anoxic condition, more than under the oxic condition. ZL/NWFM capping was effective in interrupting the release of NH_4_-N and T-N at all temperatures in this study. AC/NWFM was effective in interrupting the release of PO_4_-P and T-P at high temperatures. The increase in temperature accelerates the consumption of DO in water, which leads to the release of nutrients and greenhouse gases. The release of N and P results in algal bloom, which further deteriorates the water quality of lake sediments, particularly in terms of the DO concentration. The production of greenhouse gases from sediments increases the potential of global warming. In conclusion, ZL/NWFM and AC/NWFM capping on the sediments contaminated with N and P are suggested for breaking this vicious circle.

## Materials and Methods

### Materials

The sediment used in this study was sampled from a lake located in Anseong city, Korea, in January 2018. The sampling methods for the sediments and lake water were described in a previous study^20^. The sediments were collected using a Van Veen grab sampler, and they were homogenized by mechanical mixing before use. Lake water for the experiment was collected using a polyvinyl chloride airtight container. Subsequently, sampled water was filtered with a GF/C filter (1.2 μm pore size, Cat. No. 1822–047, Whatman, UK) and sterilized with an autoclave (HB-506, Hanbaek Scientific Co., Korea).

ZL, AC, and NWFM used in this study were supplied by local Korean companies, namely Kaya Carbon Company, Rex Material Co. Ltd, and E&H Company, respectively. Both ZL and AC had a grain size of 1.18–2.36 mm, and they were used in the experiment without further cleaning. NWFM were synthesized from polypropylene by the melt-blown method. The average pore diameter and thickness of the NWFM were 15.64 μm and 300 μm, respectively. The methods and results for the analysis of physical/chemical properties of these capping materials and their adsorption capacity for humic acid, N, and P were described in our previous studies^20^. The results obtained in previous studies are summarized in Table S1.

### Laboratory sediment incubation experiments

Laboratory sediment incubation experiments were performed by setting up nine different conditions (three different capping conditions × three different temperatures) to assess the effectiveness of sediment capping in terms of reducing release of N and P at different temperatures (4, 15, and 30 °C). The laboratory sediment incubation experiments were described in a previous study^20^. The sediment column had an internal diameter of 15 cm and a height of 25 cm, and a sampling port was placed at 9-cm height. Thirty-mL of water sample was collected through the sampling port using a 50 mL syringe. A DO probe (HI9146, Hanna Instruments, Romania) was inserted through the center of the column lid, fixed, and air tightened with silicone resin paste to prevent the evaporation of overlying water and introduction of oxygen from atmosphere to overlying water. To investigate the efficiency of capping materials at different temperatures, three different experimental systems were designed: (1) no capping (UN); (2) 3-cm thick capping of ZL under NWFM (ZL/NWFM); (3) 3-cm thick capping of AC under NWFM (AC/NWFM); all of which were placed above 5 cm of contaminated sediments at different temperatures. In the absence of capping materials, 3 cm of clean sea sand was placed below the sediments to provide equal distance between the sediment/water interface and the sampling port. A peristaltic pump (Model 7527–15, Cole-Parmer, USA) was employed to fill the sediment column with 3 L of lake water without the disturbance of sediments and the capping layer. These sediment columns were monitored for 60 days at 25 °C under airtight and light-tight conditions. Water was sampled at 1, 2, 3, 10, 17, 24, 31, 38, 45, 52, and 60 days after the start of the experiment and analyzed with respect to the environmental condition variables (including pH, EC, and ORP) and nutrient concentrations (including NH_4_-N, NO_3_-N, T-N, PO_4_-P and T-P).

### Analysis of sediments and water

The sediment water content was measured through weight loss by drying at 105 °C after 24 h. The dried sediment was then passed through a 2.0 mm sieve, and the <2.0 mm fraction was used for soil chemical analysis. pH and EC were measured using a pH/EC meter (Seven-multi S40, Mettler Toledo, Switzerland) after 10 g of soil was shaken with 50 mL of deionized water for 30 min. Organic matter (OM) was determined by loss of weight following heating in a furnace at 550 °C for 4 h. T-N was determined using the Kjeldahl digestion method, using K_2_SO_4_ and CuSO_4_ (9: 1) as catalysts. NH_3_-N was measured using the colorimetric method after extracting 5.0 g soil with 50 mL of 2 M KCl and filtering with a glass filter (No. 2, Whatman, USA), while NO_3_-N was measured by the Brucine method after extraction with 2 M KCl. T-P was determined by the ascorbic acid reduction method after thermal decomposition using perchloric acid (HClO_4_). Available phosphate content was determined using the Lancaster method. COD was measured by titration with 0.1 N Na_2_S_2_O_3_·5H_2_O after oxidizing the soil with 0.1 N KMnO_4_.

Prompt chemical analysis of water samples is assumed to reduce errors due to time delay. In the case of water samples, DO was measured using a DO meter (HI 9146, Hanna, Romania). pH and EC were measured using a pH/EC meter. COD was measured by the chromic acid method, in which samples are oxidized by K_2_Cr_2_O_7_. T-N content was analyzed with a UV spectrophotometer (Optizen POP QX, Mecasys Co., Republic of Korea) after thermal decomposition using NaOH and K_2_S_2_O_8_. NH_4_-N content was analyzed using the indophenol method and NO_3_-N was analyzed by the Brucine method. T-P was measured using the ascorbic acid reduction method after digestion with K_2_S_2_O_8_, while PO_4_-P was measured using the stannous chloride method.

### Data analysis

The flux of nutrients released from contaminated sediments to overlying water was calculated as follows^12,40^:

1 $${J}_{D}=\frac{\Delta C\times V}{A\times t},$$

where *J*_*D*_ is the flux of nutrients released from contaminated sediments during the incubation experiment (mg/m^2^·d), *ΔC* is the difference between nutrient concentrations at time *t* and initiation (mg/L), *V* is the volume of overlying water (L), *A* is the cross-sectional area of the column reactor (m^2^), and *t* is the period between sampling and the start of the incubation experiment (d).

A one-way analysis of variance (ANOVA) was carried out to compare the means of the different treatments. When significant *F*-values were detected, the differences between individual means were tested using the Tukey’s multiple range test. All statistical analyses were performed using SAS 9.4 (SAS Institute Inc., Cary, NC, USA).

## Supplementary information

Supplementary Information
