# Supplementary material for: Effect of temperature on capping efficiency of zeolite and activated carbon under fabric mats for interrupting nutrient release from sediments
Source: Sci Rep. 2019 Oct 31;9:15754. doi: 10.1038/s41598-019-52393-1 (PMC6823541; doi:10.1038/s41598-019-52393-1)
Supplement: Supplementary file 1 — Supplementary Information [file 41598_2019_52393_MOESM1_ESM.pdf]

Supplementary material

Effect of temperature on capping efficiency of zeolite and activated carbon under fabric mats for interrupting nutrient release from sediments

Seung-Hee Hong[1], Jae-In Lee[1], Chang-Gu Lee[2], Seong-Jik Park[1,*]

[1] Department of Bioresources and Rural System Engineering, Hankyong National University, Anseong, Korea
[2] Department of Environmental and Safety Engineering, Ajou University, Suwon, South Korea

* Corresponding author. S.-J.P. (*E-mail address:* parkseongjik@hknu.ac.kr)

Table S1. Physical properties and chemical composition of zeolite, activated carbon, and nonwoven fabric mats (NWFM) modified from previous studies.

		Zeolite	Activated Carbon	NWFM
Surface area (m^2/g)		52.10	1146.74	0.08
Pore volume (cm^3/g)		0.10	0.10	0.68
Pore size (nm)		3.83	3.83	15.64
Bulk density		1.04	0.476	0.111
pH		9.02	9.77	7.25
XRF Result (%)	MgO	0.28		
	Al_2O_3	11.20		
	SiO_2	62.00		
	P_2O_5	1.16		
	K_2O	13.0		
	CaO	2.42		
	TiO_2	0.77		
	Fe_2O_3	7.32		
X-ray spectrum (%)	C		93.3	94.7
	O		2.4	5.3
	S		1.7	
	Si		1.4	
	Ca		0.6	
	Fe		0.5	
	Al		0.3	
Adsorption Capacity (mg/g)	Humic Acid	2.772	125.82	86.09
	NH_4-N	19.518	3.99	2.01
	NO_3-N	2.029	2.78	1.34
	PO_4-P	2.545	1.19	0.11
